# The serum protein levels of the tPA–BDNF pathway are implicated in depression and antidepressant treatment

**DOI:** 10.1038/tp.2017.43

**Published:** 2017-04-04

**Authors:** H Jiang, S Chen, C Li, N Lu, Y Yue, Y Yin, Y Zhang, X Zhi, D Zhang, Y Yuan

**Affiliations:** 1 Department of Psychosomatics and Psychiatry, ZhongDa Hospital, Medical School of Southeast University, Nanjing, China; 2 Institute of Psychosomatics, Medical School of Southeast University, Nanjing, China; 3 Institute of Pattern Recognition and Intelligent Computing, College of Computer Science and Technology, Nanjing University of Aeronautics and Astronautics, Nanjing, China

## Abstract

Evidence demonstrates that brain-derived neurotrophic factor (BDNF) has a pivotal role in the pathogenesis of major depressive disorder (MDD). Precursor-BDNF (proBDNF) and mature BDNF (mBDNF) have opposing biological effects in neuroplasticity, and the tissue-type plasminogen activator (tPA)/plasmin system is crucial in the cleavage processing of proBDNF to mBDNF. However, very little is known about the role of the tPA–BDNF pathway in MDD. We examined serum protein concentrations in the tPA–BDNF pathway, including tPA, BDNF, tropomyosin receptor kinase B (TrkB), proBDNF and p75NTR, obtained from 35 drug-free depressed patients before and after 8 weeks of escitalopram (mean 12.5 mg per day) or duloxetine (mean 64 mg per day) treatment and 35 healthy controls using sandwich ELISA (enzyme-linked immunosorbent assay) methods. Serum tPA and BDNF and the ratio of BDNF/proBDNF were significantly lower in the MDD patients than in controls, whereas TrkB, proBDNF and its receptor p75NTR were higher. After 8 weeks of treatment, tPA, BDNF and proBDNF and the BDNF/proBDNF ratio were reversed, but p75NTR was higher than baseline, and TrkB was not significantly changed. tPA, BDNF, TrkB, proBDNF and p75NTR all yielded fairly good or excellent diagnostic performance (area under the receiver operating characteristic curve (AUC) >0.8 or 0.9). Combination of these five proteins demonstrated much better diagnostic effectiveness (AUC: 0.977) and adequate sensitivity and specificity of 88.1% and 92.7%, respectively. Our results suggest that the tPA–BDNF lysis pathway may be implicated in the pathogenesis of MDD and the mechanisms underlying antidepressant therapeutic action. The combination of tPA, BDNF, TrkB, proBDNF and p75NTR may provide a diagnostic biomarker panel for MDD.

## Introduction

Major depressive disorder (MDD) is one of the most common psychiatric disorders, with a lifetime risk of 20–25% for women and 7–12% for men.^1^ The World Health Organization suggests that depression will be the foremost contributor to the worldwide burden of disease by 2030.^2^ However, the pathophysiology of this disease is still poorly understood. Treatments are not satisfactory and are often delayed in their onset and afflicted with side effects. Recently, the neurotrophin hypothesis has received significant attention, and brain-derived neurotrophic factor (BDNF) is one of the most important neurotrophic factors that is widely measured.^3, 4, 5, 6^ The hypothesis proposes that decreased neurotrophic signaling is a major factor underlying the pathophysiology of depression, and its restoration underlie the actions of antidepressant treatment.

Studies have reported that BDNF expression levels were significantly decreased in the brains of rats exposed to stress paradigms,^7^ but they were increased by chronic tranylcypromine, sertraline, desipramine and mianserin treatment.^8^ It has also been reported that BDNF was lower in the brains of suicidal depressed patients.^9, 10^ BDNF has an important role in neuron survival, neurogenesis and synaptic plasticity.^11, 12^ BDNF arises from a glycosylation precursor, precursor BDNF (proBDNF), which is proteolytically cleaved to produce mature BDNF (mBDNF).^13^ The tissue plasminogen activator (tPA)/plasmin system is the most significant protease involved in the cleavage of proBDNF.^14, 15^ According to the ‘yin-yang neurotrophin hypothesis',^13^ mBDNF binds predominantly to tropomyosin receptor kinase B (TrkB) receptors that have anti-apoptotic properties and inhibit long-term depression. However, proBDNF, in binding preferentially to p75NTR, has opposite effects on mBDNF and favors neuronal apoptosis and long-term depression. Thus, the proteolytic processing of proBDNF by the tPA/plasminogen system represents a powerful means of regulating the direction of BDNF action that in turn could be implicated in MDD pathogenesis and in the mechanism of action of antidepressant drugs.^13, 16^ However, very little is known about the role of the tPA–BDNF pathway in depression. The diagnosis of MDD is now based on relatively subjective assessments of symptoms. The development of reliable diagnostic tests using biomarkers could aid in the diagnosis of MDD. Compared with a single marker, multiple biomarkers may reduce the impact of variation between populations and subgroups. Serum protein of BDNF and its regulators have also been reported to be involved in depression.^17, 18, 19, 20, 21^ However, the diagnostic efficacy of combining these proteins is still unknown.

The aim of this study was (i) to evaluate whether serum protein levels of BDNF and its regulators in the tPA–BDNF pathway, including tPA, BDNF, TrkB, proBDNF and p75NTR, are altered in drug-free MDD patients and to observe their putative changes during antidepressants treatment; (ii) to observe whether these proteins could serve as biomarkers for MDD diagnosis and treatment responsiveness; and (iii) to assess the associations between the clinical variables and serum protein concentrations of the above factors.

## Materials and methods

### Study population

The study was approved by the Human Participants Ethics Committee of ZhongDa Hospital of Southeast University. The trial was registered on the Chinese Clinical Trial Registry (www.chictr.org.cn) and the identifier is ChiCTR-OCH-13003133. All the subjects were given informed consent. Thirty-five inpatients with MDD (11 males and 24 females; average 43.97±13.33 years old) were recruited at the Affiliated ZhongDa Hospital of Southeast University from July 2013 to March 2015. The patients met the diagnostic criteria for MDD according to the Diagnostic and Statistical Manual of Mental Disorders-IV criteria. Before entering the study, all patients had been drug-free for at least 2 weeks. Exclusion criteria for all patients were comorbid Axis I diagnosis, including alcohol or substance abuse or dependence, or other neurological illness, including dementia or stroke. The severity of depression was assessed by two trained psychiatrists using the 17-item Hamilton Depression Rating Scale (HDRS). Thirty-five healthy controls (19 males and 16 females; average 56.74±4.59 years old) were recruited from the general community within the same time frame that the MDD patients were enrolled. They were not on medication, had no history of Diagnostic and Statistical Manual of Mental Disorders-IV Axis I or II disorder, or family history of psychiatric disorders, neurological illness, alcohol or drug abuse and dependence or other medical conditions. All controls had HDRS scores <7. We randomly administered the following antidepressant drugs to the 35 patients for 8 weeks: 20 patients received escitalopram at a mean dose of 12.5 mg per day and 15 received duloxetine at a mean dose of 64 mg per day. Response to treatment was quantified as a reduction of >50% of the HDRS score from baseline to week 8.

### Blood collection

Following an overnight fast, venous blood samples were collected into coagulant tubes between 0630 and 0800 h. Then, the blood samples were centrifuged at 3000 r.p.m. at 4 °C for 20 min and stored at −80 °C until assay. The serum samples were collected both at baseline and after 8 weeks of antidepressant treatment.

### Measurement of serum protein concentrations using enzyme-linked immunosorbent assay

We measured serum protein concentrations of tPA, BDNF, proBDNF, p75NTR and TrkB using enzyme linked immunosorbent assay (ELISA). Serum concentrations of tPA, BDNF and p75NTR were measured by ELISA kit (tPA (DTPA00; R&D Systems, Minneapolis, MN, USA); BDNF (DBD00, R&D Systems); and p75NTR (ab155436, Abcam, Cambridge, UK)) following the manufacturer's instructions. ProBDNF and TrkB concentrations were estimated using Duoset human ELISA Kit (ProBDNF: DY3175 and TrkB: DYC397; R&D Systems) combined with DuoSet ELISA Ancillary Reagent Kit (DY008; R&D Systems) following the manufacturer's instructions. All the experiments were performed in duplicate. The details of the procedure are shown in the Supplementary Data.

### Statistical analysis

The data were expressed as the mean±s.d. and analyzed with SPSS 17.0 software (IBM, Chicago, IL, USA). The protein values deviating from the mean by more than 3 s.d. were defined as outliers, which were left out. The distributions of all the variables were checked with the Kolmogorov–Smirnov test. The Chi-square test was performed on the categorical data of gender. The one-way analysis of variance and nonparametric test (Kruskal–Wallis H test) were used to compare the general characteristics, clinical and biological data between controls and ‘pre-treatment' or ‘post-treatment'. If the age, education and body mass index (BMI) were correlated with protein levels, we used multiple linear regression to make a comparison to limit the possible influence of these demographics. The analysis of proteins and HDRS scores between ‘pre-treatment' and ‘post-treatment' was conducted with paired-samples *t*-test or Wilcoxon test (paired samples). The correlation between the serum protein and the clinical variables in MDD patients were calculated by Spearman correlation. Then, we adopted the linear support vector machine to evaluate the potential diagnostic effectiveness of these proteins, controlling for the effects of gender, age, education and BMI. We performed normalization methods to process the protein levels and the demographic data, including gender, age, education and BMI. Then, we used fivefold cross-validation to evaluate the performance of the classification method. As a ‘supervised' learning algorithm, the support vector machine has been widely used to solve the problem of data classification.^22, 23^ We implemented the linear support vector machine classifier using the LIBSVM toolbox^24^ with a default parameter value. We evaluated the classification performance of these five proteins by measuring the classification accuracy, sensitivity, specificity and the area under the receiver operating characteristic curve (AUC; AUC: 0.9–1=excellent; 0.8–0.9=good; 0.7–0.8=fair; 0.6–0.7=poor; 0.5–0.6=fail). The statistical threshold was set at *P*<0.05.

## Results

### Characteristics of MDD patients and controls

The demographic variables of all subjects are shown in Table 1. There was no significant difference in gender between MDD patients and healthy controls (*X*^2^=3.68, *P*=0.053). However, there were significant differences in age (F=27.73, *P*<0.0001), education (*Z*=−4.04, *P*<0.0001) and BMI (F=7.52, *P*=0.008) between MDD patients and the controls. The HDRS scores in MDD patients were significantly decreased after 8 weeks of drug treatment (*Z*=−5.2, *P*<0.0001), but they were still higher than the controls (*Z*=−2.34, *P*=0.019). Most of the patients were responsive to 8 weeks of antidepressant treatment in this study (*n*=34), with a reduction of HDRS scores >50% only one patient was a non-responder.

### Serum levels of tPA, BDNF, TrkB, proBDNF and p75NTR

Table 1 shows the protein levels in the serum of MDD patients and controls. We found that the protein levels of tPA and BDNF were significantly lower in patients than controls (tPA: *t*=−2.82, *P*=0.006; BDNF: *t*=−3.1, *P*<0.0001). The concentrations of proBDNF, p75NTR and TrkB were significantly higher in patients compared with the controls (proBDNF: *t*=5.80, *P*<0.0001; p75NTR: *t*=9.71, *P*<0.0001; TrkB: *Z*=−3.20, *P*<0.0001). The value of BDNF/proBDNF was lower in patients than controls (*t*=5.92, *P*<0.0001).

After 8 weeks of drug treatment, serum tPA and BDNF were significantly higher in post-treatment than pre-treatment (tPA: *Z*=−2.29, *P*=0.022, BDNF: *t*=−4.82, *P*<0.0001), and they were not significantly different compared to the controls (tPA: *t*=−0.07, *P*=0.648; BDNF: F=0.01, *P*=0.933). Moreover, proBDNF levels in subjects post-treatment were significantly lower than pre-treatment (*Z*=−2.43, *P*=0.015), and this was not significantly different from the controls (*t*=0.20, *P*=0.66). p75NTR was upregulated after treatment (*t*=2.13, *P*=0.041) and even higher than controls (*t*=9.96, *P*<0.0001). There was a trend for increased TrkB in post-treatment rather than pre-treatment, although the difference did not reach statistical significance (*Z*=−1.51, *P*=0.131), and it was still higher than the controls (*Z*=−3.60, *P*<0.0001). The value of BDNF/proBDNF was upregulated by drug treatment (F=−5.07, *P*<0.0001) and it was higher than the controls (*t*=−3.87, *P*<0.0001).

### Correlations of serum protein levels with clinical variables in MDD patients

We found no significant correlations between these serum protein levels and the severity of depression (HDRS scores) in MDD patients (all *P*>0.05). Furthermore, there were no significant correlations among these serum protein levels and age, education, BMI, episodes and duration of illness (all *P*>0.05), except that proBDNF was correlated with age (rho=0.37, *P*=0.048) and education (rho=−0.59, *P*=0.001). No significant correlation was observed between changes in protein levels and reduction rates of HAMD score (tPA: rho=−0.107, *P*=0.41, BDNF: rho=−0.191, *P*=0.29, TrkB: rho=−0.341, *P*=0.08, proBDNF: rho=−0.054, *P*=0.78, p75NTR: rho=−0.038, *P*=0.84) after 8 weeks of drug treatment.

### Discriminant analysis

**Figure 1:**
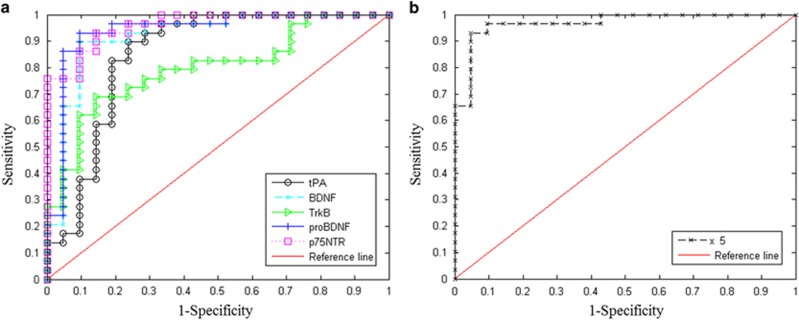
Diagnostic value of the five serum proteins. (**a**) ROC curves of each protein for MDD diagnosis. (**b**) ROC curve for the combination of these five proteins. BDNF, brain-derived neurotrophic factor; MDD, major depressive disorder; proBDNF, precursor BDNF; ROC, receiver operative characteristic; tPA, tissue-type plasminogen activator; TrkB, tropomyosin receptor kinase B.

The diagnostic value of these five proteins is shown in Table 2 and Figure 1. tPA (sensitivity=67.9%, specificity=94.1% and accuracy=81.8%) and TrkB (sensitivity=60.6%, specificity=85.0%, accuracy=73.8%) demonstrated good diagnostic effectiveness, with AUC=0.854 and AUC=0.826, respectively. In particular, BDNF, proBDNF and p75NTR showed excellent diagnostic power. The combination of these five proteins demonstrated much better diagnostic power, providing 88.1% for sensitivity and 92.7% for specificity, 0.977 for AUC and 90.6% for accuracy for MDD diagnosis.

## Discussion

In this study, we found that the dysfunction of the tPA–BDNF lysis pathway in serum was implicated in MDD pathogenesis, and it was partially reversed by antidepressant treatment. We found that serum levels of tPA and BDNF and the ratio of BDNF/proBDNF were lower in MDD patients than in controls, whereas TrkB, proBDNF and its receptor p75NTR were higher. The impairment of the tPA–BDNF cascade was partially reversed after 8 weeks of treatment, and tPA, BDNF and proBDNF and the value of BDNF/proBDNF were reversed, but p75NTR was higher than baseline, and TrkB was not significantly changed.

First, we found that BDNF was reduced in depressed patients and that these levels normalized following antidepressant treatment. The results are consistent with several previous studies,^17, 18^ strongly indicating the disruption of BDNF signaling in depression and the restoration processes induced by antidepressant treatment. This result is corroborated by meta-analyses demonstrating that BDNF levels in the brain and serum are firmly connected.^25^ Interestingly, we found that the TrkB protein is significantly higher in depression patients than in healthy controls. This result is consistent with one previous study^26^ but is contrary to several previous studies reporting that levels of TrkB were lower in the brain regions^27, 28, 29^ and serum^19^ of MDD patients. The reason for this discrepancy is not known; we speculate that it could be because of the differences in samples and methodology. An animal study also showed that repeated immobilization stress could significantly increase TrkB mRNA levels.^30^ The upregulation of TrkB may be a compensatory response to prolonged, stress-induced downregulation of BDNF in depression. To the best of our knowledge, this is the first report showing that expression of BDNF and TrkB in serum can be regulated in opposite directions. After drug treatment, there was a trend for increased trkB in post-treatment rather than pre-treatment, although the difference did not reach statistical significance. These results indicate that impaired BDNF-TrkB signaling was modulated by antidepressant treatment.

As proBDNF binding to p75NTR has opposing biological consequences to BDNF, it is essential to differentiate proBDNF from BDNF in depression. However, little information is known about the role of serum proBDNF. In this study, we found that proBDNF and its receptor p75NTR were higher in MDD patients than in the controls, suggesting a potential role of proBDNF/p75NTR signaling in the pathogenesis of depression. This result is consistent with a previous study of higher serum proBDNF and p75NTR during acute depressive episodes.^19^ Furthermore, we report for the first time that the increased proBDNF was decreased by antidepressant treatment, suggesting normalization following drug treatment.

In addition, we found that the BDNF/proBDNF ratio was decreased in depression, which is consistent with two previous studies,^19, 31^ indicating that the imbalance or the insufficient conversion of proBDNF to BDNF might have a role in major depression. In addition, another study found that prenatal stress could inhibit the proteolytic conversion of proBDNF to the BDNF in the rat hippocampus.^26^ Interestingly, we report for the first time that the decreased serum BDNF/proBDNF ratio in depression was reversed by antidepressant treatment, suggesting that the antidepressant effect might be due to the elevation of the BDNF/proBDNF ratio. Similarly, animal studies have shown that early enriched environment and physical activity have antidepressive effects that were related to the increased conversion of proBDNF to BDNF in the mouse hippocampus.^32, 33^

We found that serum tPA was decreased in depression and that this effect was reversed with drug treatment, suggesting that it was associated with MDD pathogenesis. Although conventionally known to be important for thrombosis, the tPA/plasminogen system was recently discovered to have a vital role in MDD pathophysiology by involving the cleavage process of proBDNF to mBDNF.^15, 33, 34^ Our results are consistent with previous findings that plasma tPA levels were significantly decreased during depression episodes^20, 21^ and acute mental stress.^15^ In addition, our precious study found that in plasminogen activator inhibitor-1, the major inhibitor of tPA, the protein expression was increased in the prefrontal cortex and hippocampus of rats showing depression-like behaviors and in the serum of depressed patients.^35^ Therefore, the decreased activity of the tPA/plasminogen system may cause the imbalance or the insufficient conversion of proBDNF to BDNF, resulting in impaired BDNF signaling that could cause impaired neural plasticity in depression. In turn, the high levels of tPA in post-treatment patients may have promoted a higher rate of cleavage of proBDNF, contributing to the lower levels of proBDNF and the higher levels of BDNF and the BDNF/proBDNF ratio observed in this study.^31^

Moreover, the results of discriminant analysis showed that tPA, BDNF, TrkB, proBDNF and p75NTR all yielded fairly good or excellent diagnostic performance, and the combination of these five proteins demonstrated much better diagnostic effectiveness. These results suggested that tPA, BDNF, TrkB, proBDNF and p75NTR in the tPA–BDNF pathway in serum all should be considered as a biomarker for MDD diagnosis, and the combination of these five proteins may provide a potential biomarker panel as a diagnostic test for MDD.

Altered peripheral BDNF levels have also been reported in other common psychiatry disorders, including bipolar disorder and schizophrenia.^36, 37^ The initial symptom of bipolar disorder and schizophrenia overlaps with the depressive features of MDD. An MDD misdiagnosis in bipolar disorder or schizophrenia patients is commonly associated with inappropriate antidepressant treatment and can worsen the outcome for bipolar disorder or schizophrenia patients. We will further study these serum protein levels of the tPA–BDNF pathway in these mental disorders to determine the utility of this biomarker panel for differentiating MDD from other psychiatric disorders and identifying subgroups of patients with MDD.

There are some limitations to the present study. First, the number of patients with MDD in our study was small. Second, almost all the patients were responsive to 8 weeks of antidepressant treatment in this study, so we are unable to analyze whether these proteins in the tPA–BDNF pathway could serve as biomarkers for treatment responsiveness. Finally, the age and education of the subjects were not well matched in our study. These preliminary results need further study in larger and well-matched cohorts.

Collectively, our results suggest that the tPA–BDNF lysis pathway may be implicated in the pathogenesis of MDD and the mechanisms underlying antidepressant therapeutic action. A single protein above in the tPA–BDNF pathway in serum should be considered a biomarker for MDD diagnosis. The combination of these five proteins may provide a potential biomarker panel as a diagnostic test for MDD.

## Figures and Tables

**Table 1:** The demographic and neuropsychological characteristics of major depressive disorders (mean±s.d.)

	*Controls (*n*=35)*	*MDD (*n*=35)*	
		*Pre-treatment*	*Post-treatment*
Age (years)	56.74±4.59^a^	43.97±13.33**	—
Gender (male/female)	19/16^b^	11/24	—
Education (years)	8.51±2.44^c^	12.03±4.47**	—
BMI	25.20±3.16	22.97±3.63**	—
Duration of illness (months)	—	27.82±52.29	—
Episodes	—	1.34±0.68	—
HDRS_17_	2.23±1.97^c^	21.57±4.63**	7.23±5.12^##^^f^
tPA (pg ml^−1^)	2856.09±1641.81^d^	1749.28±750.20**	2062.23±993.82^#^^f^
BDNF (pg ml^−1^)	23420.16±8525.71^d^	17380.21±5161.24**	23597.39±8910.28^##^^e^
TrkB (pg ml^−1^)	582.65±662.18^c^	642.85±206.47**	724.59±291.48**^f^
proBDNF (pg ml^−1^)	50.00±36.17^d^	101.18±32.85**	83.80±41.66^#^^f^
p75NTR (pg ml^−1^)	18.17±5.42^d^	28.98±3.15**	30.34±4.21**^#^^e^
BDNF/proBDNF	651.62±413.28^d^	175.74±63.21**	325.36±160.81**^##^^e^

Abbreviations: BDNF, brain-derived neurotrophic factor; BMI, body mass index; HDRS_17_, 17-item Hamilton Depression Rating Scale; MDD, major depressive disorders; proBDNF, precursor BDNF; tPA, tissue-type plasminogen activator; TrkB, tropomyosin receptor kinase B.

a ANOVA (analysis of variance) analysis, controls vs ‘Pre-treatment' or ‘Post-treatment'.

b Chi-square test, controls vs ‘Pre-treatment' or ‘Post-treatment'.

c Mann–Whitney *U*-test, controls vs ‘Pre-treatment' or ‘Post-treatment'.

d Multiple linear regression analysis.

e Paired-samples *t*-test, ‘Before treatment' vs ‘After treatment'.

f Wilcoxon test (paired samples), ‘Before treatment' vs ‘After treatment'.

Several abnormal values were removed for BDNF, proBDNF, TrkB, p75NTR and the value of BDNF/proBDNF. After removing the abnormal values, the remaining number of cases showed as follows: BDNF (33 MDD and 35 controls), proBDNF (29 MDD and 33 controls), TrkB (27 MDD and 34 controls), p75NTR (32 MDD and 31 controls), BDNF/proBDNF (27 MDD and 33 controls). Finally, 21 MDD patients and 29 healthy controls had all the five proteins. Compared with controls, *P<0.05, **P<0.01. Compared with ‘Pre-treatment', ^#^*P*<0.05, ^##^*P*<0.01.

**Table 2:** Diagnostic value of these five proteins.

*Name*	*AUC*	*Accuracy (%)*	*Sensitivity (%)*	*Specificity (%)*
tPA	0.854	81.8	67.9	94.1
BDNF	0.934	86.7	84.7	89.5
TrkB	0.826	73.8	60.6	85.0
proBDNF	0.933	88.6	83.8	92.9
p75NTR	0.967	85.5	80.0	90.2
× 5	0.977	90.6	88.1	92.7

Abbreviations: AUC, area under the receiver operating characteristic curve; BDNF, brain-derived neurotrophic factor; proBDNF, precursor BDNF; tPA, tissue-type plasminogen activator; TrkB, tropomyosin receptor kinase B; × 5, combination of tPA, BDNF, TrkB, proBDNF and p75NTR.
