# Supplementary material for: The serum protein levels of the tPA–BDNF pathway are implicated in depression and antidepressant treatment
Source: Transl Psychiatry. 2017 Apr 4;7(4):e1079–. doi: 10.1038/tp.2017.43 (PMC5416686; doi:10.1038/tp.2017.43)
Supplement: Supplementary Information [file tp201743x1.doc]

The detection details of each protein were different, following the manufacturer’s instructions. The kit protocols can be found on the official sites by looking up the kit numbers:

tPA (DTPA00; R&D Systems); the URL of the manufacturer’s instructions: <https://resources.rndsystems.com/pdfs/datasheets/dtpa00.pdf>;

BDNF (DBD00, R&D Systems); the URL of the manufacturer’s instructions: <https://resources.rndsystems.com/pdfs/datasheets/dbd00.pdf>;

TrkB (DYC397; R&D Systems); the URL of the manufacturer’s instructions: <https://resources.rndsystems.com/pdfs/datasheets/dyc397.pdf>；

Pro-BDNF (DY3175, R&D Systems); the URL of the manufacturer’s instructions: <https://resources.rndsystems.com/pdfs/datasheets/dy3175.pdf>).

p75NTR (ab155436, Abcam); the URL of the manufacturer’s instructions: <http://www.abcam.cn/Human-NGF-R-ELISA-Kit-CD271-ab155436.pdf>；

Duoset human ELISA Kit of ProBDNF and TrkB concentrations were combined with DuoSet ELISA Ancillary Reagent Kit (DY008; R&D Systems); the URL of the manufacturer’s instructions: <https://resources.rndsystems.com/pdfs/datasheets/dy008.pdf>
